# Pentadecanoic Acid-Releasing PDMS: Towards a New Material to Prevent *S. epidermidis* Biofilm Formation

**DOI:** 10.3390/ijms251910727

**Published:** 2024-10-05

**Authors:** Caterina D’Angelo, Serena Faggiano, Paola Imbimbo, Elisabetta Viale, Angela Casillo, Stefano Bettati, Diana Olimpo, Maria Luisa Tutino, Daria Maria Monti, Maria Michela Corsaro, Luca Ronda, Ermenegilda Parrilli

**Affiliations:** 1 Department of Chemical Sciences, University of Naples “Federico II”, Complesso Universitario Monte S. Angelo, Via Cintia 4, 80126 Naples, Italy; caterina.dangelo@unina.it (C.D.); paola.imbimbo@unina.it (P.I.); angela.casillo@unina.it (A.C.); diana.olimpo@unina.it (D.O.); tutino@unina.it (M.L.T.); mdmonti@unina.it (D.M.M.); corsaro@unina.it (M.M.C.); 2 Department of Food and Drug, University of Parma, Parco Area delle Scienze 23/A, 43124 Parma, Italy; 3 Institute of Biophysics, National Research Council (CNR), Via G. Moruzzi 1, 56124 Pisa, Italy; stefano.bettati@unipr.it (S.B.); luca.ronda@unipr.it (L.R.); 4 Department of Medicine and Surgery, University of Parma, Via Volturno 39, 43125 Parma, Italy; elisabetta.viale@unipr.it

**Keywords:** pentadecanoic acid, antibiofilm, PDMS, ABIFAB, *S. epidermidis*, surface coating

## Abstract

Microbial biofilm formation on medical devices paves the way for device-associated infections. *Staphylococcus epidermidis* is one of the most common strains involved in such infections as it is able to colonize numerous devices, such as intravenous catheters, prosthetic joints, and heart valves. We previously reported the antibiofilm activity against *S. epidermidis* of pentadecanoic acid (PDA) deposited by drop-casting on the silicon-based polymer poly(dimethyl)siloxane (PDMS). This material exerted an antibiofilm activity by releasing PDA; however, a toxic effect on bacterial cells was observed, which could potentially favor the emergence of resistant strains. To develop a PDA-functionalized material for medical use and overcome the problem of toxicity, we produced PDA-doped PDMS by either spray-coating or PDA incorporation during PDMS polymerization. Furthermore, we created a strategy to assess the kinetics of PDA release using ADIFAB, a very sensitive free fatty acids fluorescent probe. Spray-coating resulted in the most promising strategy as the concentration of released PDA was in the range 0.8–1.5 μM over 21 days, ensuring long-term effectiveness of the antibiofilm molecule. Moreover, the new coated material resulted biocompatible when tested on immortalized human keratinocytes. Our results indicate that PDA spray-coated PDMS is a promising material for the production of medical devices endowed with antibiofilm activity.

## 1. Introduction

Advances in biomedical devices are ingenious and essential in healthcare, saving millions of lives, but, at the same time, their interaction with the human body can introduce new vulnerabilities and new disease conditions [1]. Indeed, microbial biofilm formation on medical devices paves the way to device-associated infections with high morbidity and mortality [2,3,4,5]. As the population ages, there is an increasing need to restore and maintain the quality of biomedical devices. Therefore, the problem of biofilm-associated infections is expected to become more and more prominent [6,7].

The biofilms elude antibiotics facilitating the persistence of infections and the development of antimicrobial resistance (AMR) [8,9]; the microbial contamination of medical devices generates niches capable of favoring emerging pathogens, previously considered saprophytic species. This is the case of *Staphylococcus epidermidis*, one of the most common strains involved in medical device contamination [10,11]. Indeed, due to its normal presence on human skin, it can colonize numerous indwelling medical devices, such as intravenous catheters, prosthetic joints, and heart valves [12]. The ability of *S. epidermidis* to form biofilm is central to the infection process and represents its major virulence determinant [13]. Therefore, the research of new antibiofilm molecules able to counteract this emerging pathogen is becoming urgent [14,15,16,17,18,19,20,21].

Among other approaches, antibiofilm molecules produced by cold-adapted bacteria proved promising in this field [22,23,24,25,26]. The Antarctic bacterium *Pseudoalteromonas haloplanktis* TAC125 produces a long chain fatty aldehyde, pentadecanal [27], able to interfere with the *S. epidermidis* AI-2/LuxS quorum sensing system and to strongly affect the biofilm formation process [23,25,27]. Although pentadecanal is very promising as antibiofilm molecule, it could easily lose its activity since aldehydes are highly reactive [28]. Therefore, pentadecanal derivatives were designed and the corresponding acid, pentadecanoic acid (PDA), showed good antibiofilm properties against *S. epidermidis* [28]. Moreover, an antibiofilm coating through the adsorption of PDA on a poly(dimethyl)siloxane (PDMS) surface has been proposed [29], being PDMS commonly applied in biomedical implants due to its favorable biological and material properties [30,31]. As a matter of fact, this polymer is frequently used for the manufacture of medical devices, such as phonatory and mammary prostheses, catheters and drug-delivery systems, due to its physiological inertness, high blood compatibility, low toxicity, and good thermal and oxidative stability [30,32,33]. It has to be pointed out that, even though PDMS is a good candidate for medical applications due to its favorable physico-chemical properties, in some cases surface or bulk modifications are necessary, as this silicone-based polymer is prone to bacterial adhesion and eventually biofilm formation [32,34].

In Ricciardelli et al. [29], the PDA coating on the PDMS surface was performed by drop-casting. This coating strongly reduced the formation of the staphylococcal biofilm but also induced a toxic effect on the bacterial cells probably due to a non-homogeneous distribution of PDA on the surface after drop-casting [29]. Since the killing action on bacterial cells increases the emergence of antibiotic resistance, this toxicity should be avoided in systems aimed at preventing biofilm formation. Moreover, to implement the use of PDA, a complete description of the process of its release is necessary to evaluate the drug-release capacity and long-term efficacy of the system.

This research aims to push towards the concrete use of PDA functionalized materials in the medical field by developing different coating methods to obtain a uniform coating of the PDMS surface with PDA and by setting up a strategy to assess the kinetics of PDA release. To accomplish an exhaustive characterization of the release kinetics, we made use of a free fatty acids fluorescent probe, ADIFAB (AcryloDan labelled Intestinal Fatty Acid Binding protein), able to sense their presence even at low concentrations (in the micro- to nanomolar range) [35,36,37,38,39,40]. Finally, the biocompatibility of the new PDA-coated PDMS material was assessed on a cell-based model to establish its safeness on humans. 

## 2. Results

### 2.1. Optimizing Antibiofilm Coatings of PDMS: Spray-Coating and Incorporation of PDA in PDMS

In a previous study, coating of PDA on a poly(dimethyl)siloxane (PDMS) surface was performed by drop-casting [29], whereas, in this work, two different methods were used to obtain antibiofilm PDMS surfaces: the incorporation of PDA into PDMS during the polymerization (PDA-incorporated PDMS), and the spray-coating upon PDMS polymerization (PDA spray-coated PDMS).

Regarding PDA spray-coated PDMS, a solution of PDA in acetone was airbrushed on PDMS by a commercial airbrush using a pressure of 15 PSI, and the PDA surface concentration obtained was 0.48 mg/cm^2^. In the case of PDA-incorporated PDMS, PDA in acetone was mixed with a polymer mixture to obtain PDMS with PDA at the final concentration of 5 mg/mL and a superficial concentration of 0.96 mg/cm^2^. For both techniques, acetone was specifically selected as the carrier solvent due to the solubility of PDA in acetone. Furthermore, the high volatility of acetone allows for fast evaporation at room temperature, ensuring the complete removal of the solvent and leaving the functional PDMS material with relatively low toxicity, a feature desirable for healthcare applications.

**Figure 1:**
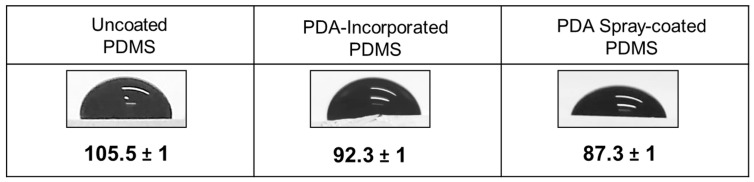
Effect of PDA on PDMS hydrophobicity. Pictures of water droplets and water contact angle measurements of uncoated PDMS, PDA-incorporated PDMS, and PDMS spray-coated with PDA. Each data represents the mean ± SD of three independent measurements.

As a first characterization, the hydrophobicity of these new antibiofilm PDMS surfaces was evaluated by water contact angle (WCA) measurements (Figure 1). The contact angle of uncoated PDMS was 105.5 ± 1 degrees, whereas it decreased for PDA-incorporated in PDMS and PDA spray-coated PDMS resulting respectively 92.3 ± 1 and 87.3 ± 1 degrees. The reduction of the contact angle indicates a reduced hydrophobicity of the surface. PDA incorporation in PDMS and spray-coating gave a similar effect on the hydrophobicity of the PDMS surface compared to PDA drop-casting, which was also reported to increase the hydrophilicity of the surface, due to the presence of the ionic carboxylic group of the pentadecanoic acid [29].

The FTIR spectra revealed that no covalent bonds were established between PDMS and PDA since no shift of the carbonyl signal at 1739 cm^−1^ of PDA was observed (Supplementary Materials, Figure S1). In addition, the signal of PDMS at 2154 cm^−1^ decreased in neither PDA spray-coated nor PDA-incorporated PDMS infrared spectra, thus indicating that PDA was not covalently grafted into PDMS [41].

### 2.2. Evaluation of Biofilm Formation on Coated PDMS in a Flow Cell System

**Figure 2:**
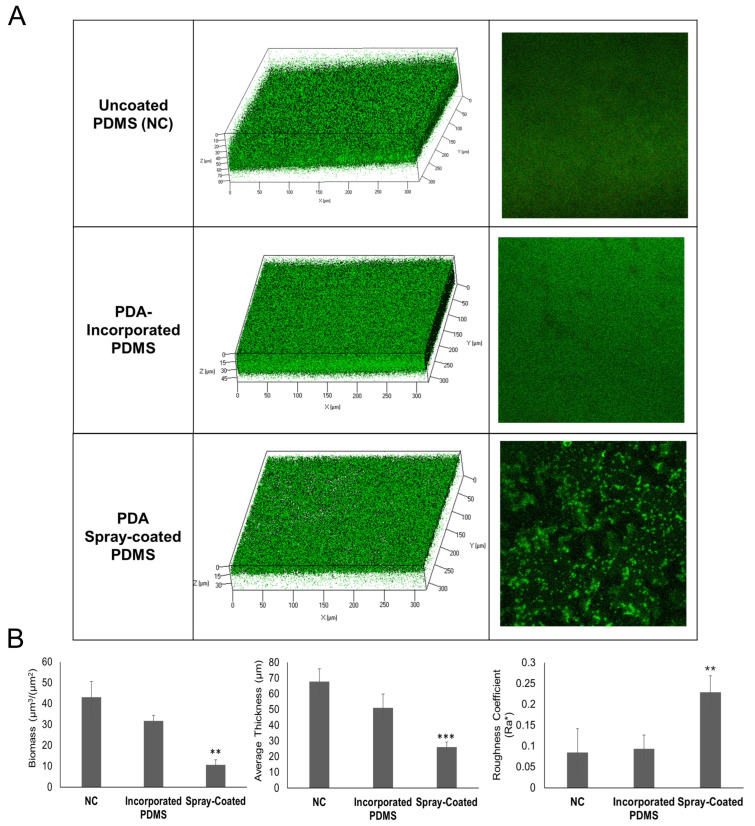
Evaluation of biofilm formed in convertible flow cells on PDMS surfaces. (**A**) CLSM analysis of *S. epidermidis* RP62A biofilms formed on uncoated PDMS (NC), PDA-incorporated PDMS and PDA spray-coated PDMS. Bi-dimensional and three-dimensional biofilm structures were obtained using the LIVE/DEAD^®^ Biofilm Viability Kit. (**B**) COMSTAT quantitative analysis of biomass (µm^3^/µm^2^), average thickness (µm), and roughness coefficient (Ra*) of biofilms formed on uncoated PDMS (NC), PDA-incorporated PDMS and PDMS spray-coated with PDA. Differences in mean values were compared to the uncoated PDMS (NC) values and considered significant when *p* < 0.05 (** *p* < 0.01, *** *p* < 0.001) according to the Student’s *t*-test.

The biofilm formation of *S. epidermidis* RP62A on uncoated PDMS, PDA-incorporated PDMS, and PDA spray-coated PDMS was evaluated in a flow cell system. In detail, the system was assembled by connecting a container for the input solution buffer, a peristaltic pump (with a flow rate of 160 μL/min), a bubble-trap (to prevent air bubbles that disturb the coating), a convertible flow cell containing uncoated PDMS (NC), PDA-incorporated PDMS or PDA spray-coated PDMS, a container for the output solution buffer, and connecting tubing. Briefly, a volume of culture was flowed for 2 h into the convertible flow cells, connected in parallel to the flow system. Then, the biofilms were grown for 24 h and the biofilm structures and cell viability were analyzed by confocal laser scanning microscopy (CLSM) (Figure 2). Bi-dimensional and three-dimensional biofilm structures were obtained using live/dead staining, in which viable cells appear green and dead (cell membrane damaged) bacteria appear in red. CLSM analysis (Figure 2A) demonstrated that the biofilm formed on the NC was thick and had a compact structure, and the biofilm formed on PDA-incorporated PDMS appeared similar to NC. Noteworthy, the biofilm obtained on PDA spray-coated PDMS was less homogeneous and thinner compared to NC and PDA-incorporated PDMS. These results were confirmed also by COMSTAT 2.1 image software package analysis (Figure 2B). The PDA spray-coated PDMS allowed a reduction of biofilm biomass and thickness, and an increase in the roughness coefficient.

The roughness coefficient is a dimensionless factor that provides the measure of the thickness variation of a biofilm and is used as an indirect indicator of biofilm heterogeneity. The analysis revealed that the spray-coating treatment resulted in an inhomogeneous and unstructured biofilm. Moreover, the spray-coating technique avoided the dead cell zone previously recovered by drop casting [29].

### 2.3. PDA Release Kinetics from PDMS Functionalized by Spray-Coating in a Fixed-Volume Setup

Since PDA spray-coated PDMS was proven to reduce biofilm formation, it was subjected to further analyses to assess PDA release kinetics. ADIFAB, a fluorescent probe able to bind free fatty acids, was used to quantify free PDA in solution. ADIFAB is a commercially available probe constituted by recombinant rat intestinal fatty acid binding protein (I-FABP) covalently conjugated to a fluorescent probe (acrylodan). When a fatty acid binds to ADIFAB, acrylodan is displaced from the fatty acid binding site, exposing acrylodan to a more polar environment (in aqueous buffers) and causing a change in the fluorescence emission of the probe, thus allowing a quantitative estimation of free fatty acids (FFAs) concentration. The probe does not bind to fatty acids in micelles. I-FABPs have evolved with the specific function of recognizing FFAs, so in principle it allowed us to have a specific probe for the fatty acid present in our experiments, i.e., PDA. ADIFAB can bind fatty acids of different lengths; K_d_ values for myristic acid (14C) and palmitic acid (16C) at different temperatures are reported in the product manual (at 25 °C, K_d_s are 3.07 μM and 0.28 μM, respectively). To measure the concentration of PDA (15C) in solution, we had to validate its binding to ADIFAB and to determine the K_d_ for PDA, which resulted to be 1.14 μM at 25 °C, indicating, as reasonable, an intermediate affinity between myristic and palmitic acid. Given that the lowest limit for FFA determination with ADIFAB is about 1 nM [35], this probe enabled the measurement of very low FFA concentrations, overcoming the limit of other alternative quantification methods.

**Figure 3:**
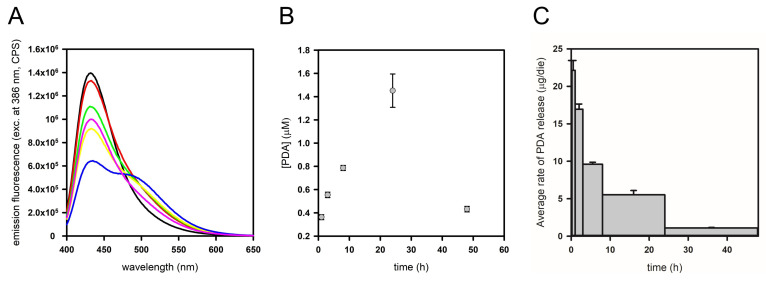
Analysis of PDA release from PDA spray-coated PDMS using the fluorescent probe ADIFAB. (**A**) ADIFAB fluorescence emission spectra of PDA spray-coated PDMS in agitation over time: ADIFAB in fresh PBS, time = 0 (black line), in PBS buffer after 1 h (red line), 3 h (green line), 8 h (yellow line), 24 h (blue line) and 48 h (pink line). (**B**) PDA concentration in the PBS samples. (**C**) Average rates of PDA release.

To assess PDA release kinetics, pieces of PDA spray-coated PDMS were placed at the basis of cylindric containers (i.e., wells of a 6-well culture plate). A fixed volume of PBS was added, and the plate was put under agitation on an orbital shaker. At given times (1, 3, 8, 24, and 48 h), PBS was replaced with fresh buffer and the recovered solution was used to determine the amount of released PDA for each given time interval. This buffer exchange ensured that the release rate was not limited by PDA solubility and concentration in the medium. In Figure 3, ADIFAB spectra (A), the corresponding free PDA concentrations (B) and the average rates of PDA release (C) are reported for each time interval.

It can be noticed that the amount of free PDA present in the PBS buffer tends to increase up to 24 h (Figure 3B), while slightly decreasing after 48 h. Finally, an average PDA release rate was calculated for each time interval, by dividing the amount (μg) of free PDA in the PBS collected at each time by the incubation time, which is the time for which the same buffer was in contact with the PDMS support (Figure 3C). It appears that the average release rate is faster in the first hours of the experiment and tends to diminish throughout the experiment. A cumulative analysis of the amount of released PDA was also performed (Supplementary Materials, Figure S2). In these conditions, after 48 h about 9 μg of PDA were released from the PDMS support. The total amount of released PDA increases over time and the rate of release tends to diminish at longer times.

### 2.4. Long-Term Efficacy of the Antibiofilm Coating on Biofilm Formation in a Flow Cell System

Biofilm growth in the flow cells system was also tested on PDA spray-coated PDMS previously exposed for 21 days to PBS flow. As a control, a cell with uncoated PDMS (NC) and fresh PDA spray-coated PDMS were used. Staphylococcal biofilms were grown for 24 h and the biofilm structures and viability of the bacteria were analyzed by confocal laser scanning microscopy (CLSM), as previously reported.

**Figure 4:**
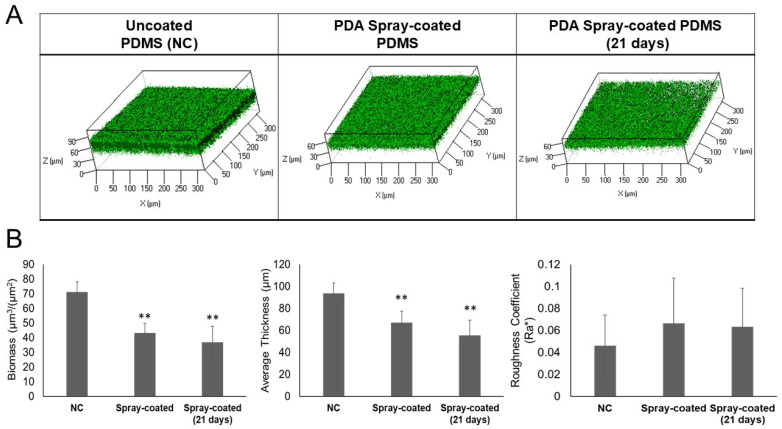
Long-term efficacy of the PDA spray-coated PDMS. (**A**) CLSM analysis of *S. epidermidis* RP62A biofilms formed in convertible flow cells on uncoated PDMS (NC) (left panel), PDA spray-coated PDMS freshly coated with pentadecanoic acid (central panel) and PDA spray-coated PDMS exposed to a constant PBS buffer flow for 21 days (right panel). Three-dimensional biofilm structures were obtained using the LIVE/DEAD^®^ Biofilm Viability Kit. (**B**) COMSTAT quantitative analysis of biomass (µm^3^/µm^2^), average thickness (µm), and roughness coefficient (Ra*) of biofilm on uncoated PDMS (NC), PDA spray-coated PDMS freshly coated with PDA and PDA spray-coated PDMS exposed to a constant PBS buffer flow for 21 days. Differences in mean values were compared to the uncoated PDMS (NC) values and considered significant when *p* < 0.05 (** *p* < 0.01) according to the Student’s *t*-test.

As shown in Figure 4, CLSM analysis demonstrated a strong capability of the coating to reduce the biofilm formation of *S. epidermidis* RP62A, even after 21 days of exposure to PBS, thus proving its long-term efficacy.

### 2.5. Evaluation of the Drug-Release Capability of PDA Spray-Coated PDMS in a Cell Flow System and Biocompatibility of Functionalized Material

**Figure 5:**
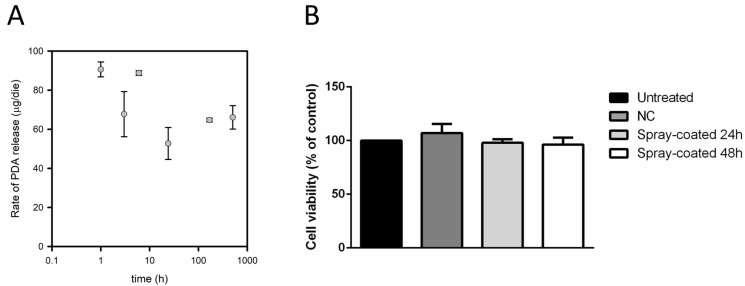
Release capability and biocompatibility of PDA spray-coated PDMS. (**A**) Rate of PDA release over time. The instantaneous rate of PDA release at 1, 3, 6, 24 h, 7 days, and 21 days of cell-flow experiments is reported. (**B**) Effect of PDMS on cell viability. Immortalized human keratinocytes were incubated in the presence of uncoated PDMS (dark grey bar), and PDA spray-coated PDMS for 24 or 48 h (light grey and white bars, respectively). Cell survival was expressed as the percentage of viable cells in the presence of different PDMS-based materials under test compared with control cells (i.e., untreated cells, black bar). Values are given as means ± SD (*n* ≥ 3). Differences in mean values were compared to the uncoated PDMS (NC) values and considered significant when *p* < 0.05 according to the Student’s *t*-test. The data were not significantly different compared to the control.

Experiments in a cell flow system demonstrated the long-term (21 days) efficacy of PDA spray-coated PDMS in inhibiting biofilm formation. To characterize the PDA release kinetics in dynamic conditions during a 21 days-long experiment under continuous flux, the study was performed using a cell flow system setup. The support had a surface of 5.25 cm^2^ and was loaded with 0.48 mg of PDA/cm^2^, with a total amount of PDA of 2.5 mg. Samples of 3 mL PBS buffer were collected during the experiment and free PDA was quantified by using ADIFAB (Supplementary Materials, Figure S3). The concentrations of free PDA measured were rather similar (in the range 0.8–1.5 μM) at different times (1, 3, 6, 24 h, 7 days, and 21 days) (Figure S3). This indicated that the concentration of released PDA in a cell flow system continued throughout the experiment. Even after 21 days of continuous buffer flow, PDA is released at a concentration close to 1 μM, suggesting that PDA spray-coated PDMS acts as a reservoir able to deliver efficient amounts of the antibiofilm molecule (Figure 5A). It must be noticed that the flux of buffer over the PDMS support was continuous, thus the rate of release can be expressed as an instantaneous rate, rather than being an average rate for a given time interval, as for the first setup used. Although data fitting to a linear or exponential equation resulted in undetermined parameters, overall, data indicate that the rate of PDA release over 21 days is in the range 50–90 μg/die, allowing the release of an effective amount of antibiofilm molecule throughout the experiment. Knowing the total volume of PBS flowed over the PDMS support (about 5.4 L), it was possible to estimate that the total amount of PDA released after 21 days is about 2 mg, hence, about 80% of the total loaded amount. This result supports the evidence that the gradual release of PDA from spray-coated PDMS can provide an antibiofilm activity for up to 21 days of buffer flow. Also, it can be noticed that the release rate was higher in the cell flow system compared to the fixed volume setup, indicating that the continuous flux of fresh buffer over the surface facilitated PDA release.

To further investigate a possible use of the PDA spray-coated PDMS for medical devices, its biocompatibility was evaluated. The effect on cell viability was evaluated on immortalized human keratinocytes (HaCaT). 24 h after seeding, cells were incubated with uncoated PDMS, or with PDA spray-coated PDMS for 24 or 48 h. At the end of incubation, cell survival was determined by the MTT assay. As shown in Figure 5B, no effect on cell viability was observed when cells were incubated with either uncoated PDMS or PDA spray-coated PDMS, independently from the time of the incubation (24 and 48 h), thus supporting its biocompatibility. No direct correlation between contact angle, roughness and biocompatibility was found considering uncoated, PDA-incorporated and spray-coated PDMS.

## 3. Discussion

The prevention of biofilm formation on medical device surfaces is a critical area of research due to the significant hurdles that biofilm-associated infections pose [42]. The resilience of bacteria within biofilms against antibiotics, disinfectants, and biocides makes biofilm treatment exceptionally challenging, underlining the importance of preventive measures [43]. Surface coatings capable of inhibiting biofilm formation offer a promising solution [44,45,46]: various strategies have been explored, including coatings releasing antibacterial agents such as antibiotics, peptides, nanoparticles, or nitric oxide [47,48,49,50], as well as the use of specialized surface textures, polymer grafting, and biofilm-dispersing enzymes [51,52,53,54]. However, each strategy presents its unique obstacles. Surfaces with incorporated antimicrobial agents may lose efficacy over time and contribute to antimicrobial resistance, while those grafted with PEG or zwitterionic polymers often exhibit transient effects due to bacterial protein and surfactant adsorption [51]. Despite their potential, further developments are needed to enhance the durability of these surfaces.

Recent efforts have turned towards modifying PDMS, a silicon-based elastomer widely used in biomedical applications [30] due to its unique properties including biocompatibility, resistance to biodegradation, chemical inertness, favorable mechanical properties, gas permeability, optical transparency, and ease of fabrication [55].

Various methods have been developed for modifying PDMS such as the adsorption of coating materials onto its surface [55,56]. However, achieving an ideal antibiofilm surface requires ensuring that the modification process does not compromise the polymer’s mechanical properties or biocompatibility. Additionally, when the surface is coated with a bioactive molecule, it is essential to maintain controlled kinetics of release and effective concentrations, and to ensure long-term efficacy, resistance to in vivo degradation and limited capacity to induce microbial resistance [57]. Moreover, the modification process should be straightforward, scalable, and cost-effective for practical application.

In this context, an effective antibiofilm surface was previously developed by modifying PDMS through the adsorption of pentadecanoic acid (PDA) onto its surface [29]. In Ricciardelli et al. [29], the PDA coating on the PDMS surface was achieved through the drop-casting method. The proposed coating strategy has proven to be able to form weak interactions between PDA and PDMS, leading thus to a release of the antibiofilm molecule over time. The poor water solubility of the PDA is crucial in obtaining a slow release of the compound in an aqueous solution [29]. Nevertheless, the amount of PDA released allows a strong reduction in *S. epidermidis* biofilm formation. Indeed, PDA is active at very low concentrations since it acts as a quorum sensing modulator [27,28]. This coating was demonstrated to significantly reduce the formation of the staphylococcal biofilm, while also exerting an unwanted toxic effect on bacterial cells.

To solve the cell-death problem, in the present study two different methods were employed for the developing of antibiofilm PDMS surfaces: the incorporation of PDA into PDMS during the polymerization (PDA-incorporated PDMS), and the PDA deposition on PDMS surface using the spray-coating technique (PDA spray-coated PDMS). The antibiofilm performance of the two new surfaces was assessed by growing *S. epidermidis* RP62A in a flow cell system and evaluating the biofilm structures and cell viability by CLSM. The results highlighted that the biofilm formed on PDA-incorporated PDMS material had a similar structure compared to the biofilm formed on uncoated PDMS (control), showing that PDA-incorporated PDMS had no antibiofilm activity. This result could be due to the reduced diffusion of PDA from the PDMS surface. Generally, PDMS is inherently impermeable to water, but some molecules can modify the swelling of the polymer making it more permeable and allowing an easier release of the molecules entrapped in this material, as observed in the case of salicylic acid [58]. It is reasonable to hypothesize that the presence of PDA within the polymeric matrix of PDMS could not change the local permeability of the polymer to water, preventing the release of PDA from PDMS. Moreover, the results obtained from the FTIR experiments allowed us to exclude the formation of covalent linkages between PDMS and PDA for both the tested surfaces. The biofilm developed on PDA spray-coated PDMS appeared less homogeneous and thinner compared to PDA-incorporated PDMS. Notably, this result was obtained despite the PDA surface concentration used in the case of PDA spray-coated PDMS was half of that of PDA-incorporated PDMS.

These results were confirmed by COMSTAT analysis, revealing that the spray-coating treatment resulted in the formation of an inhomogeneous and unstructured biofilm. Interestingly, the spray-coating technique, compared to the drop-casting method [29], preserved *S. epidermidis* cells viability. This key feature makes the new antibiofilm surface highly attractive for medical device applications because the absence of any killing action on bacterial cells reduces the emergence of antibiotic resistance. Indeed, the development of resistance is a problem for currently available antibiotic coatings. For example, one study indicated that gentamicin-resistant staphylococci were recovered from gentamicin-loaded beads implanted in a patient after arthroplasty, highlighting the potential risk of resistance development due to exposure to biomaterial-associated antimicrobials [59]. Given that *S. epidermidis* has emerged as a significant causative agent of nosocomial and medical device-related infections [60], and considering its ability to acquire resistance to multiple antibiotics, antibiofilm strategies that avoid the development of resistant mutants are crucial to prevent *S. epidermidis* infections.

The results presented here demonstrated that, after exposure to PBS under a constant flow for 21 days, PDA spray-coated PDMS continued to exhibit a clear antibiofilm effect; this is essential for the efficacy and durability of the antibiofilm coating. Additionally, the procedure used for constructing this surface is simple and can be easily adapted and scaled up for industrial production lines, facilitating the direct translation of the technology for the functionalization of PDMS.

PDA can effectively reduce the biofilm formation of *S. epidermidis* RP62A at very low concentrations (12.5 μg/mL) [28]. In this work, the efficacy and prolonged activity of the novel antibiofilm surface indicated a gradual release of PDA over time, ensuring its continuous availability at an active concentration. A quantitative description of the kinetics of PDA release from PDMS is crucial to setting up an extended-release drug delivery device and achieving a favorable biological response. The low concentration of PDA required for the antibiofilm activity requires a very sensitive detection method to accurately quantify the release of PDA from the PDMS surface.

The investigation of the kinetics of PDA release using the fluorescent probe ADIFAB provided valuable insights into the drug-release capacity of the PDA spray-coated PDMS system. By monitoring the release of PDA over time, its sustained efficacy in preventing biofilm formation can be clearly explained, and essential information for optimizing the design of antibiofilm coatings and ensuring their long-term effectiveness in clinical settings can be obtained. The kinetics of PDA release were evaluated firstly using a fixed volume of PBS, measuring the release under orbital agitation. With this setup, the amount of free PDA tends to increase up to 24 h while slightly decreasing after 48 h. The concentrations of the FFA are in the low micromolar range and the total amount of released PDA increases over time, reaching about 9 μg after 48 h. When release kinetics were monitored using a continuous flux setup, the detected release rates were higher compared to the fixed volume setup. Experiments in a cell flow system demonstrated the long-term (21 days) efficacy of PDA spray-coated PDMS in inhibiting biofilm formation. The determination of PDA concentration in PBS samples collected from the cell flow apparatus indicated that PDA release from PDMS is continuous over 21 days, with a range of rates between 50 and 90 μg/die, allowing the release of an effective amount of antibiofilm molecule throughout the experiment. Considering the amount of loaded PDA and the total volume of PBS fluxed over the PDMS plate, it can be calculated that after 21 days about 80% of the PDA was released, suggesting that the antibiofilm activity of the PDA coating could potentially be exerted for even longer periods of time.

Although the biocompatibility of pentadecanoic acid was previously demonstrated, the biocompatibility of the antibiofilm surface using immortalized human keratinocytes was evaluated to assess the potential use of PDA-spray coated PDMS in biomedical applications. The assay revealed that the PDA coating permits cell viability showing no toxicity effect on tested human cells.

## 4. Materials and Methods

### 4.1. Bacterial Strains and Growth Conditions

*S. epidermidis* RP62A, a reference strain isolated from an infected catheter (ATCC collection no. 35984), was grown at 37 °C in brain heart infusion broth (BHI, OXOID, Basingstoke, UK). Overnight pre-cultures were transferred to fresh medium for main cultures inoculation. Then, *S. epidermidis* cells from overnight main cultures were harvested by centrifugation at 5000× *g* for 10 min at 4 °C, washed twice with fresh phosphate-buffered saline (PBS, 10 mM potassium phosphate and 150 mM NaCl, pH 7.4) and then resuspended in fresh PBS to the desired bacterial concentration.

### 4.2. Preparation of the Antibiofilm PDMS Coatings

#### 4.2.1. Spray-Coating

PDMS surfaces were prepared using SYLGARD^®^ 184 silicone elastomer kit (Dow Corning Corporation, Midland, MI). PDMS substrates were fabricated by mixing base to curing agent to a ratio 10:1 (*w*/*w*). For the use of PDMS in the convertible flow cells, the polymer mixture was poured into the bottom of the flow cell, degassed until all air was removed and then cured at 65 °C for at least 5 h. PDMS surfaces were then sterilized by UV for 20 min. To obtain the PDA spray-coated PDMS, PDA (Sigma Aldrich, Inc., St. Louis, MO, USA) was used. Briefly, 500 µL of a 5 mg/mL PDA solution in acetone was deposited by spray onto the PDMS surface and dried at room temperature under sterile conditions until complete solvent evaporation.

#### 4.2.2. Incorporation of PDA on/in PDMS

For the PDA-incorporated PDMS, the polymer mixture (prepared as described in the previous paragraph) was mixed in the presence of PDA in acetone. Then, 1 mL of the mixture was poured into the bottom of the flow cell, and the polymerization was carried out as previously described.

### 4.3. Contact Angle Measurements

Advancing type contact angles with ultrapure water on PDMS with or without the antibiofilm coatings were measured with a locally manufactured contour monitor using the sessile drop technique [61]. On each sample, at least five droplets were placed at different positions and the images of the droplets were taken directly after 40 s from deposition. Results of three separately prepared surfaces with or without antibiofilm coatings were averaged.

### 4.4. Fourier Transform Infrared Spectroscopy Analysis (FTIR)

PDMS and PDA spectra were recorded by an FT/IR instrument (JASCO-4700 Tokio, Japan) and 128 scans interferogram was collected with a variable path length cell and KBr windows. For PDA analysis, 1 mg of PDA was combined with 100 mg of dry KBr. The mixture was then pressed and dried at 60 °C for 24 h before analysis. The spectra were recorded at a straight baseline of 400–4000 cm^−1^.

### 4.5. Antibiofilm PDMS Coating Effect on S. epidermidis Biofilm Formation in a Flow Cell System

The effect of PDA sprayed or incorporated in PDMS on *S. epidermidis* RP62A biofilm was evaluated using a flow cell method. The experiment was performed using convertible flow cells (Stovall Life Science, Inc., Greensboro, NC, USA), assembled according to the manufacturer’s instructions. The single chamber (7.7 cm^3^, 24 mm × 40 mm × 8 mm) has a detachable, re-attachable top, which allows the polymerization of the PDMS directly into the flow chamber. Culture medium or buffer flowed through the cell at a controlled flow rate of 160 μL/min using an Ismatec IPC 4 Peristaltic Pump (Cole–Parmer GmbH, Wertheim, Germany). The flow system was kept free of air bubbles using a bubble trap, which created a low positive pressure with medium or buffer flow, thus mitigating undesirable peristaltic pulsation in liquid delivery to the flow cell. A bacterial suspension of *S. epidermidis* RP62A in a solution of BHI 2% (*v*/*v*) in PBS (0.2 OD/mL) was flowed into three convertible flow cells: (1) a cell with PDA spray-coated PDMS; (2) a cell with PDA-incorporated PDMS; (3) a cell with uncoated PDMS (NC). In the beginning, PBS was circulated through the system for 1 h, then the bacterial suspension flowed in flow cells for 2 h to allow bacterial adhesion, and finally, non-adherent cells were removed, by washing with sterile PBS for 30 min. Finally, fresh medium (BHI 50% (*v*/*v*) in PBS) was circulated for 24 h through the system to allow biofilm formation on the PDMS surfaces. Biofilms were analyzed by confocal laser scanning microscopy (CLSM).

For the long-term efficacy analysis of PDA spray-coated PDMS, the bacterial suspension of *S. epidermidis* RP62A in a solution of BHI 2% (*v*/*v*) in PBS (0.2 OD/mL) was flowed into three convertible flow cells with: (1) PDA spray-coated PDMS exposed for 21 days to PBS, (2) fresh antibiofilm spray-coated PDMS and (3) uncoated PDMS (NC).

### 4.6. Confocal Laser Scanning Microscopy Analysis

For confocal laser scanning microscopy (CLSM) analysis, the bacteria viability in the biofilms was determined by FilmTracer™ LIVE/DEAD^®^ Biofilm Viability Kit (Molecular Probes, Invitrogen, Carlsbad, CA, USA), containing the SYTO^®^ 9 green fluorescent nucleic acid stain (10 mM) and propidium iodide (PI), the red-fluorescent nucleic acid stain (60 mM), which was injected with a syringe into the convertible flow cell, without removing them from the flow system, and incubated for 20–30 min at room temperature, protected from light. Then, fresh PBS was flowed to remove the excess stain. All microscopic observations and image acquisitions were performed with a confocal laser scanning microscope (LSM700-Zeiss, Jena, Germany) equipped with an Ar laser (488 nm), and a He-Ne laser (555 nm). Images were obtained using a 20×/0.8 objective. The excitation/emission maxima for these dyes are 480/500 nm for SYTO^®^ 9 stain and 490/635 nm for propidium iodide. Z-stacks were obtained by driving the microscope to a point just out of focus on both the top and bottom of the biofilms. Images were recorded as a series of .tif files with a file depth of 16 bits.

The collected CLSM Z-stack images (saved as OME-TIFF) were analyzed with COMSTAT software package a plugin (Comstat 2.1) to ImageJ. The COMSTAT software package was used to determine the different variables describing biofilm including the biovolume (µm^3^ µm^−2^), average thicknesses (µm), and roughness coefficient (Ra*).

### 4.7. Determination of PDA Release Kinetics from PDMS

PDA release from PDA spray-coated PDMS was measured using two different experiment layouts. The first setup consists of soaking the PDMS support in a fixed volume of buffer under orbital agitation, while the second setup corresponds to the cell flow system described above.

For release experiments in a fixed volume of buffer, PDMS supports having a surface of 1.75 cm^2^ spray-coated with PDA at a superficial concentration of 0.48 mg/cm^2^ were posed at the basis of a 6-well culture plate and covered with 10.5 mL of PBS. The plate was kept under agitation using an orbital shaker. At specific time points (1, 3, 8, 24, and 48 h), the buffer was recovered and substituted with the same volume of fresh PBS. The amount of free PDA released in the PBS buffer at the end of each incubation time was measured using ADIFAB, a fluorescent probe able to bind free fatty acids [35,36,37,38,39,40] (FFA Science, San Diego, CA, USA). As a preliminary step, the K_d_ of ADIFAB for PDA was experimentally determined according to the manufacturer’s specifications and then was used to determine released PDA concentrations in the samples. For each sample, 0.2 μM ADIFAB was added in a cuvette containing 700 μL of PBS sample and a fluorescence emission spectrum upon excitation at 386 nm was acquired in the range 400–700 nm. Spectra were acquired in duplicates using a FluoroMax-3 fluorometer (HORIBA-Jobin–Yvon, Edison, NJ, USA).

The concentration of released PDA was also determined using ADIFAB in samples collected from a flow cell system. The experiment was carried out as described in the section above on PDA spray-coated PDMS. PBS samples with a volume of 3 mL (the flux was 180 μL/min) were collected after 1, 3, 6, 24 h, 7 days, and 21 days. Spectra were acquired in duplicates.

### 4.8. The Antibiofilm PDMS Coating Biocompatibility Assay

Immortalized human keratinocytes (Innoprot, Derio, Spain) were cultured in Dulbecco’s modified Eagle’s medium (DMEM) (Sigma-Aldrich, St Louis, MO, USA) supplemented with 10% fetal bovine serum (HyClone, Logan, UT, USA), antibiotics and 2 mM L-glutamine. Cells were grown in a 5% CO_2_ humidified atmosphere at 37 °C and seeded in 24-well plates at a density of 8 × 10^4^ cells per well. After 24 h, uncoated PDMS and PDA spray-coated PDMS were added to cells for 24 and 48 h. At the end of incubation, cell viability was assessed by the MTT (3-(4,5-dimethylthiazol-2-yl)-2,5-diphenyltetrazolium bromide) assay. Cell survival was expressed as the percentage of viable cells in the presence of the materials under test compared to the untreated cells. Each sample was tested in three independent analyses, each carried out in triplicate.

### 4.9. Statistics and Reproducibility of Results

The significance of differences between the mean values was calculated using a twotailed Student’s *t*-test and *p* < 0.05 was considered significant.

## 5. Conclusions

This study not only introduces spray-coating as a new coating technique for PDA, developing promising antibiofilm surfaces that reduce the risk of emergence of resistance, but also offers valuable insights into the release kinetics of the PDA antibiofilm molecule from PDMS surfaces by using a protein-based fluorescent probe. A description of the release kinetics of the material was essential for predicting its performance over time, a crucial feature for its further application in medical devices. It was demonstrated that the concentration of the antibiofilm compound released over time remains between 0.8 and 1.5 μM in a flow-system, and about 80% of the total amount of PDA is released after 21 days, ensuring sustained effectiveness and long-term durability of the newly developed surface. Moreover, the biocompatibility of PDA spray-coated PDMS was also demonstrated. With their strong antibiofilm properties and biocompatibility, the coatings developed in this work hold great promise for the improvement of long-term indwelling devices. Further exploration of their applications in complex biological environments and validation through in vivo studies remain the next steps of investigation.

## Data Availability

Data is contained within the article and Supplementary Materials.

## Supplementary Materials

The following supporting information can be downloaded at: https://www.mdpi.com/article/10.3390/ijms251910727/s1.
