# Supplementary material for: Pentadecanoic Acid-Releasing PDMS: Towards a New Material to Prevent S. epidermidis Biofilm Formation
Source: Int J Mol Sci. 2024 Oct 5;25(19):10727. doi: 10.3390/ijms251910727 (PMC11476977; doi:10.3390/ijms251910727)
Supplement: Supplementary file 1 [file ijms-25-10727-s001.zip › ijms-3164623-supplementary.pdf]

Supplementary Materials

Pentadecanoic Acid-Releasing PDMS: Towards A New Material to Prevent *S. epidermidis* Biofilm Formation

Caterina D'Angelo [1], Serena Faggiano [2,3]*, Paola Imbimbo [1], Elisabetta Viale [4], Angela Casillo [1], Stefano Bettati [3,4], Diana Olimpo [1], Maria Luisa Tutino [1], Daria Maria Monti [1], Maria Michela Corsaro [1], Luca Ronda [3,4] and Ermenegilda Parrilli [1]*

1 Department of Chemical Sciences, University of Naples "Federico II", Complesso Universitario Monte S. Angelo, Via Cintia 4, 80126 Naples, Italy

2 Department of Food and Drug, University of Parma, Parco Area delle Scienze 23/A, 43124 Parma, Italy

3 Institute of Biophysics, CNR, Via G. Moruzzi 1, 56124 Pisa, Italy;

4 Department of Medicine and Surgery, University of Parma, Via Volturno 39, 43125 Parma, Italy

* Correspondence: Serena Faggiano: serena.faggiano@unipr.it; Ermenegilda Parrilli: erparril@unina.it

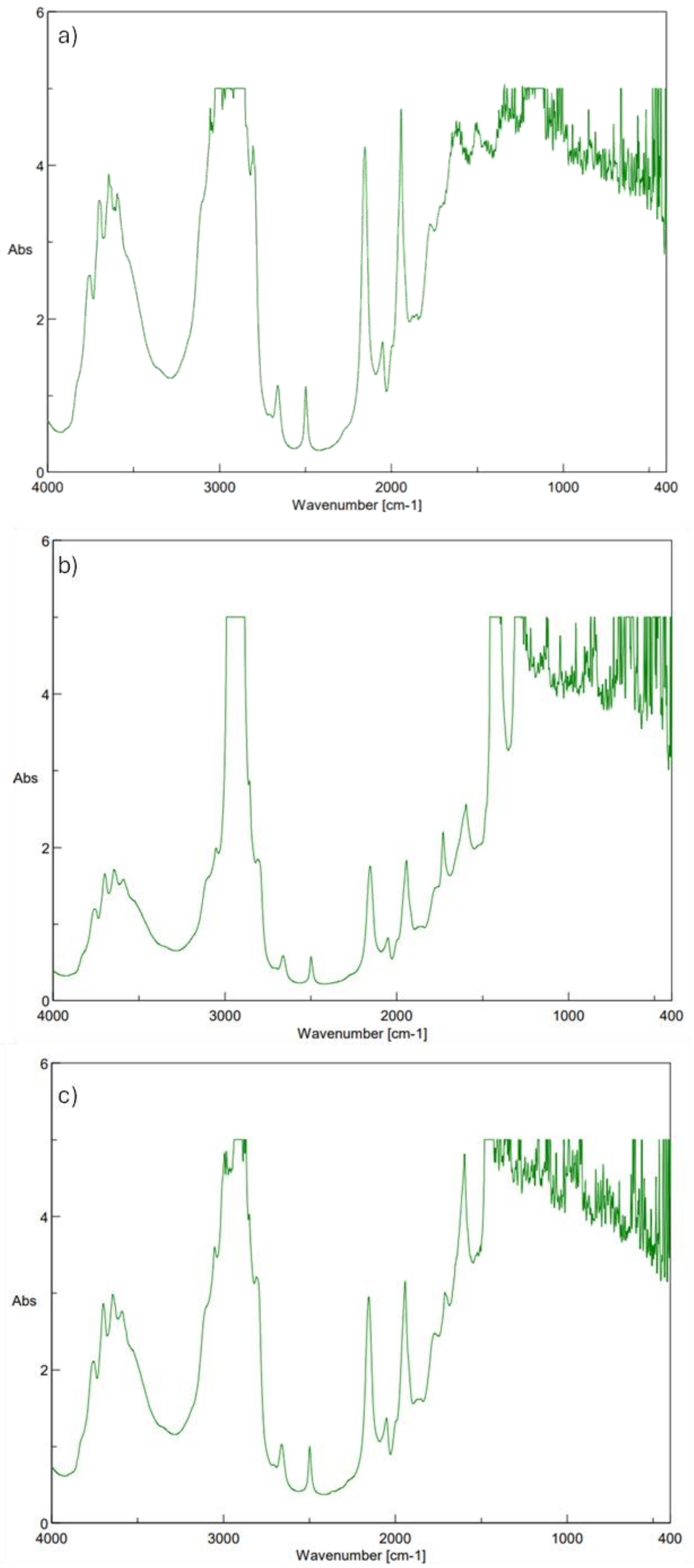

Figure S1. FTIR spectra of a) uncoated PDMS; b) PDA incorporated-PDMS, and c) PDMS spray-coated with PDA.

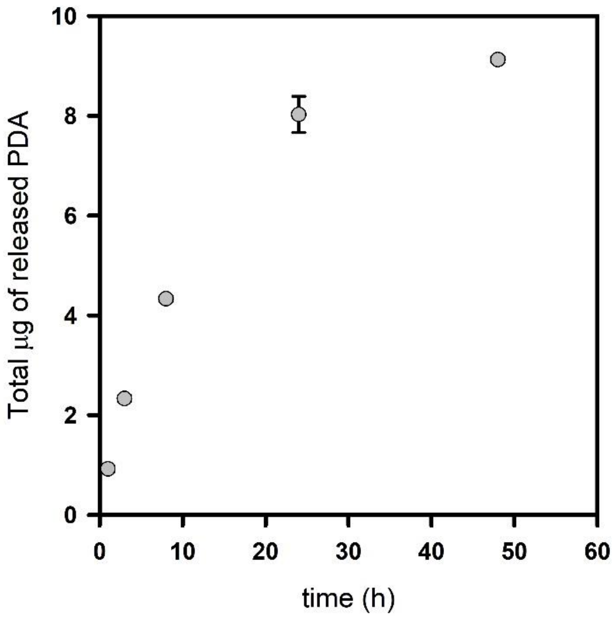

Figure S2. Total amount of PDA released in the fixed-volume experiment.

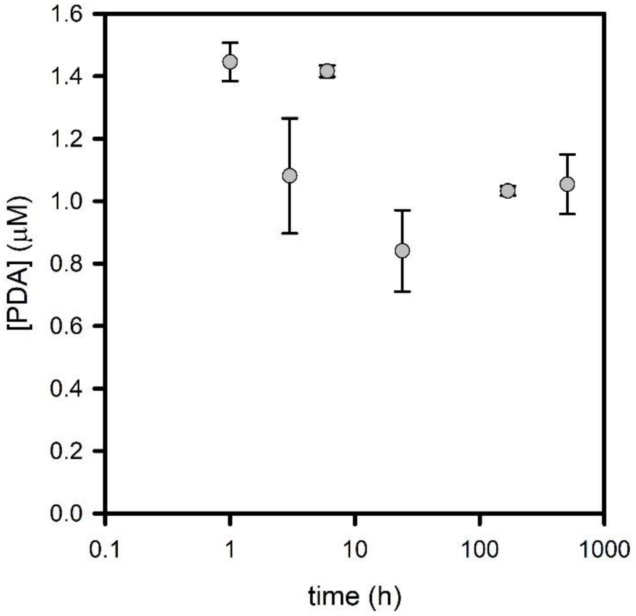

Figure S3. Analysis of PDA release from PDA spray-coated in dynamic condition. PDA concentration in the 3 ml PBS samples after 1 h, 3 h, 6 h, 7 days and 21 days of PBS continuous flux over PDA spray-coated PDMS support.
